# The hypotensive effect of salt substitutes in stage 2 hypertension: a systematic review and meta-analysis

**DOI:** 10.1186/s12872-020-01347-x

**Published:** 2020-02-27

**Authors:** Sadegh Jafarnejad, Hamed Mirzaei, Cain C. T. Clark, Mohsen Taghizadeh, Armin Ebrahimzadeh

**Affiliations:** 1 Research Center for Biochemistry and Nutrition in Metabolic Diseases, Kashan, University of Medical Sciences, Kashan, IR Iran; 2 Centre for Sport, Exercise, and Life Sciences, Coventry University, Coventry, UK

**Keywords:** Hypertension, Salt substitutes, Systolic blood pressure, Diastolic blood pressure, Meta-analysis

## Abstract

**Background:**

Hypertension (HTN) is a ubiquitous risk factor for numerous non-communicable diseases, including cardiovascular disease and stroke. There are currently no wholly effective pharmacological therapies for subjects with HTN. However, salt substitutes have emerged as a potential therapy for the treatment of HTN. The aim of the present study was to assess the effect of salt substitutes on reducing systolic blood pressure (SBP) and diastolic BP (DBP), following a meta-analysis of randomized controlled trials.

**Methods:**

Studies were found via systematic searches of the Pubmed/Medline, Scopus, Ovid, Google Scholar and Cochrane library. Ten studies, comprised of 11 trials and 1119 participants, were included in the meta-analysis.

**Results:**

Pooled weighted mean differences showed significant reductions of SBP (WMD − 8.87 mmHg; 95% CI − 11.19, − 6.55, *p* < 0.001) and DBP (WMD − 4.04 mmHg; 95% CI − 5.70, − 2.39) with no statistically significant heterogeneity between the 11 included comparisons of SBPs and DBPs. The stratified analysis of trials based on the mean age of participants showed a significant reduction in the mean difference of SBP in both adults (< 65 years old) and elderly (≥65 years old). However, the DBP-lowering effect of salt substitutes was only observed in adult patients (WMD − 4.22 mmHg; 95% CI − 7.85, − 0.58), but not in the elderly subjects.

**Conclusions:**

These findings suggest that salt-substitution strategies could be used for lowering SBP and DBP in patients with stage 2 HTN; providing a nutritional platform for the treatment, amelioration, and prevention of HTN.

## Background

Hypertension (HTN) is characterized by a diastolic blood pressure (DBP) (≥90 mmHg) and a systolic blood pressure (SBP) (≥140 mmHg) [1]. Recently, HTN has been asserted to be the leading cause of global disability-adjusted life years [2]; whilst more than 25% of adults are diagnosed with HTN, globally, and it is predicted that by 2025, 29% (1.56 billion) of the adult population will be affected [3]. New insights into the pathogenesis of HTN has suggested that a variety of risk factors contribute to the disease and may be modulated by various cellular and molecular mechanisms [4, 5]. Such mutations have been putatively linked to HTN pathogenesis; specifically, mutations of *Cyp11b1* and 11β-hydroxylase are associated with the progression of HTN, albeit in animal models [4, 5].

Cardiotonic steroids (CTSs) are a branch of hormones mechanistically related to natriuresis, the process of sodium excretion in the urine, and are believed to play an essential role in the pathogenesis of HTN, and exert impact via interaction with Na/K-ATPase, which regulates renal salt handling and help maintain the salt-sensitivity of blood pressure (BP) [6]. In addition to genetic predisposition, diet represents a major factor in the occurrence and progression of HTN. Indeed, among dietary supplements, salt substitutes have been purported to hold a potentially pivotal role in the manifestation and progression of HTN.

Additionally, it has been reported that aging is directly related to the severity of HTN; where it has been shown that after the age of 60 years, the prevalence of stage 2 HTN equates to 48.8% in individuals aged 60–79 years, and 63% those aged over 80 years, respectively [7]. Therefore, achieving a suitable control in elderly women is difficult [8]. Given that 62% of cardiovascular disorders, and 49% of ischemic heart disease, are associated with elevated BP, efficacious dietary interventions are highly sought-after. The Food and Agriculture Organization of the United Nations and World Health Organization have advocated that the consumption of less than 5 g of salt per day as a preventative measure against HTN; whilst further research has asserted that a decrease in salt consumption would likely result the reduction of global incidence of HTN, cardiovascular disease, and stroke related deaths [9]. A meta-analysis of randomized controlled trials, conducted by Peng et al., documented the impact of salt substitution on BP [10], where six cohorts from 5 articles (1974 subjects) were included; demonstrating that salt substitutes elicit significant ameliorative effects on SBP and DBP. Moreover, significant heterogeneity was observed for both SBP and DBP [10]. Notwithstanding the positive contribution made in [10], numerous limitations were present, including high heterogeneity, low sample size and limited included studies. Given such limitations exist, in addition to the potential for salt substitutes to aid in the amelioration or prevention of stage 2 HTN among adults and the elderly, the aims of the present study were; firstly, to provide an updated systematic review and meta-analysis of the hypotensive effects of salt substitutes, as manifest in randomized controlled trials (RCTs) in subjects with stage 2 HTN, and secondly, perform additional analyses investigating the effect of potential modulators which may interfere with the BP-lowering effect of salt substitutes, including participants age, duration of administration, and quality of studies.

## Methods

### Search strategy and selection of studies

The Preferred Reporting Items for Systematic Reviews and Meta-Analyses (PRISMA) guidelines were adopted to perform this systematic review and meta-analysis [11]. Initially, a systematic search of the electronic databases; Pubmed/Medline™, Scopus™, Ovid™, Google Scholar™ and Cochrane library™, up to December 2018 for studies relating to the main intervention and outcome, was conducted. In addition, we reviewed the reference lists of relevant original and previous review articles in order to identify further eligible studies. The following search terms were employed: “salt substitution” OR “salt substitute” OR salt OR sodium OR potassium, in combination with; “Blood pressure” OR “Hemodynamic parameters” OR “BP” OR “SBP” OR “Systolic blood pressure” OR “DBP” OR “Diastolic blood pressure”. No restrictions in published languages were imposed. In the case of insufficient data, we contacted the authors of the studies to request the necessary information.

Clinical trials investigating the effects of salt substitutes on blood pressure were selected for the meta-analysis if they satisfied the following inclusion criteria: being a RCT of either parallel or cross-over design, participants were diagnosed with stage 2 HTN (SBP ≥ 140 mmHg or DBP ≥ 90 mmHg) according to the recent American College of Cardiology/American Heart Association (ACC/AHA) updated guidelines [12], investigating the effects of salt substitutes, in any form, on blood pressure, reporting either blood pressure levels or mean change of both intervention and control groups before and after the trials. Exclusion criteria were: 1) low quality of study (less than two on the Jadad scale); 2) lack of a control group for salt substitute, 2) inadequate reporting data on SBP or DBP in intervention and control groups or data for calculating the mean change of outcomes.

### Data extraction and quality assessment

Two authors independently screened the data and excluded those not of relevance, and possible discrepancies were resolved through discussion with a third author. We extracted the following characteristics of each eligible study: first author’s name, study design, study location, sample size of intervention and control groups, age, follow-up duration, dosage and ingredients of salt and salt substitute, clinical condition and stage of hypertension of participants, baseline SBP and DBP levels, and quality of trials.

We assessed the quality of the included study by using the Jadad scale, that ranges from 0 to 5, with the following descriptions: (1) randomization (one point for stating random allocation and additional point for appropriate description of the method), (2) blinding (one point for stating the blindness of the trial and one additional point if the method of blinding was appropriate), and (3) reporting of participant withdrawals (one point if the outcome of all participants is known). The studies with the total score of ≥3 considered as high-quality trials.

### Statistical analysis

Results were expressed as the weighted mean difference (WMD) in systolic and diastolic blood pressure with 95% confidence intervals (CI). We calculated the net changes in outcomes in both intervention and control groups, as the differences between mean values before and after treatments in parallel trials. In crossover trials, we measured the net changes as the differences in the post treatment values of each group. We calculated SD values in the studies with no reported SD, by using the standardized method of Follman [13]. The heterogeneity between the studies was assessed by using the I^2^ test and Cochrane’s Q test at by which *P* < 0.05 or I^2^ > 50% was identified as heterogeneous. A random effects model was utilized if significant heterogeneity was evident. Otherwise, the fixed-effects model was used. We used stratified analysis to detect any possible influences of several modulators, including; clinical condition and stage of hypertension of participants, baseline systolic and diastolic blood pressure and duration of intervention. We also conducted a sensitivity analysis by omitting a single study and re-calculating the effect size to explore its influence on the overall effect size. We explored the potential publication bias by using Funnel plot, Begg’s rank correlation test and Egger’s weighted regression test.

All analyses were conducted using Review Manager Software (Review Manager 5.3; Cochrane Collaboration, Oxford, England) and Comprehensive Meta-Analysis (version 3.2; Biostat). A *P* value of less than 0.05 was considered as statistically significant in the present meta-analysis.

## Results

### Study selection

**Fig. 1:**
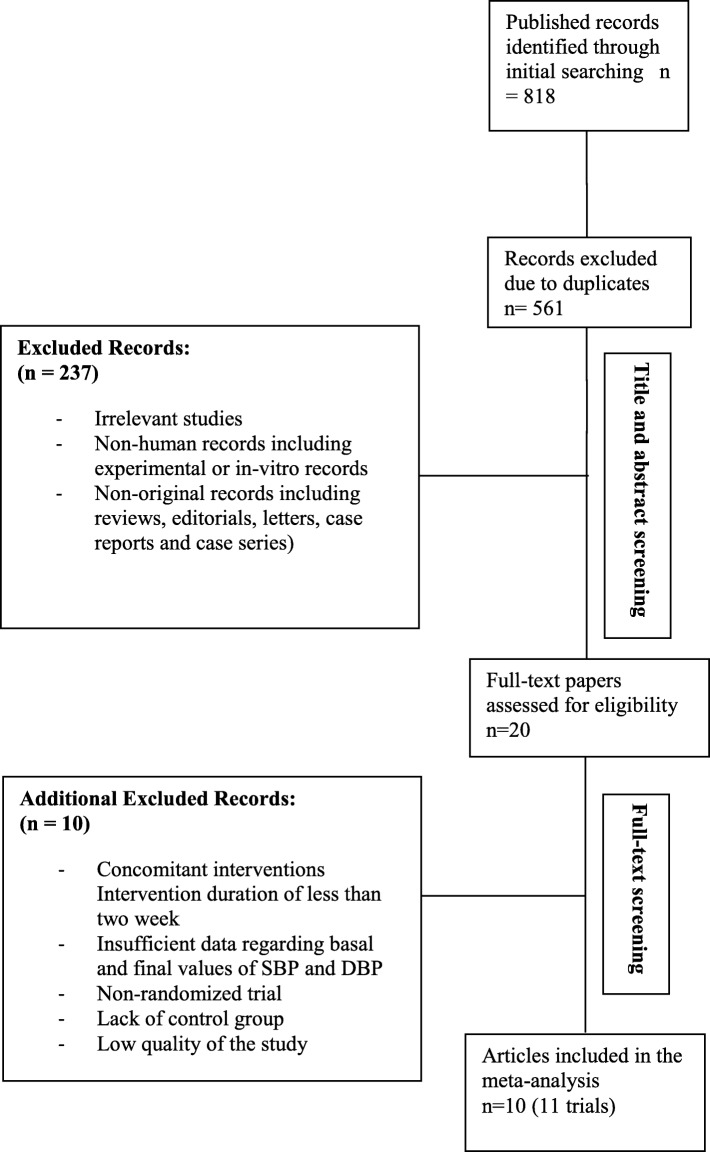
Flow diagram of literature search of the present meta-analysis

An overview of the selection process is shown in Fig. 1. The initial literature search identified 818 studies. After removal of duplicates, non-relevant papers, unavailable full-text papers, and non-original studies, including reviews, letters, editorials and case reports, 20 studies were fully reviewed, and another 10 studies were excluded because they did not meet the inclusion criteria for the meta-analysis. The reasons for the exclusion were: concomitant interventions, intervention duration of less than 8 days, insufficient data regarding baseline and final values of SBP and DBP, lack of control group and inappropriate design including low quality of the study. Yang et al. [14] investigated the effect of salt substitutes on different subsets separated by various characteristics of participants and according to the Cochrane Handbook for Systematic Reviews of Interventions [15], each arm was considered as an independent trial in the present meta-analysis. Finally, ten studies, with 11 trials and 1119 participants were included in the present meta-analysis [16–24].

### Study characteristics

Characteristics of included trials are shown in Table 1. The included trials enrolled 546 male and female participants in intervention and 573 participants in control groups (total participants of 1119), with a mean age ranging between 39.5 and 67.8 years. These 11 included trials were published between 1986 to 2018, of which five were conducted in China [14, 21, 22, 24], two in Brazil [17, 25], and one in Finland [20], France [26], Italy [27] and the UK [28], respectively. The intervention duration ranged between 8 days and 24 months. All trials used common salt (NaCl) as the salt type for control groups [14, 20–22, 24–28], except for Barros et al. who used 390 mg of sodium and 25 mcg of iodine per gram of salt [17]. The included trials used a combination of NaCl, KCl and MgSo_2_/MgSo_4_ as salt substitutes. Other micronutrients, such as calcium, folic acid, and trace minerals were used as co-ingredients of salt substitutes. Sample sizes ranged from 5 to 238, with 546 total participants in the intervention groups and 573 in the control groups, respectively. The mean SBP of intervention groups at baseline varied between 136.2 and 174.1 mmHg, and the mean DBP between 80.6 and 102.2 mmHg.

**Table 1:** Detailed description of included studies

Author	Year	Design of studies	Country	No. of Subjects in case group	No. of controls	Age of intervention group (mean)	Follow-up Duration (week)	Clinical Condition	Salt substitute	Salt type	Baseline BP	Quality	sex	method of measurement
Allaert	2013	a randomized, placebo-controlled, double-blind, clinical trial crossover study.	France	21	19	58.6(Total)	8 weeks	Stage 2 of hypertension	NaCl + Chitosan 3%	NaCl	149.2/93.4	3	m/f	Blood pressure was measured using homologated automated digital sphygmomanometers. The BP cuff was placed around the upper left arm which was supported at the level of the heart while the subject sat in a chair. Blood pressure was measured after a 10-min rest and preferably at three different times separated by 3-min intervals. The average of the three measurements was calculated.
Arzilli	1986	Randomized controlled trial	Italy	5	5	39.5(Total)	0.25 months	Stage 2 of hypertension	k/Na salt	Common salt [N/A]	> 95		m/f	NS
Barros	2015	Single-blind randomized controlled trial	Brazil	19	16	55.5(total participants)	4 weeks	Stage 2 of hypertension	130 mg of sodium, 346 mg of potassium and 44 mcg ofiodine for per gram light salt	390 mg of sodium and 25 mcg of iodine per gram salt	142.95/89.79	5	m/f	Casual BP was measured by the researcher. And home blood pressure use in this study
Gilleran	1996	a randomised blind controlled parallel study.	United kingdom	20	20	62.5	3 months	Stage 2 of hypertension and Type II diabetic	50% NaCl, 40% KCL, 10% Mg2+	Ordinary Table salt [N/A]	Sbp = 163.2	NS	m/f	NS
Pereira	2005	randomized, double-blind	Brazil	12	10	47.5(Total)	12 week	Stage 2 of hypertension	50% sodium chloride and 50% potassium chloride	NaCl100%	136.2/93.8	4	m/f	Systolic and diastolic blood pressure values during medical consultation were determined by the auscultatory method. Measurements were made with the cuff placed covering the two thirds on the upper arm with the patient sitting, after 5 min at rest, and the values of systolic and diastolic pressure
Sarkkinen	2011	Randomized, double-blind, placebo-controlled	Finland	22	23	57	8 week	Stage 2 of hypertension	50% sodium chloride (NaCl), 25% potassium chloride(KCl) and 25% magnesal; magnesium ammonium potassiumchloride, hydrate [Mg4K(NH4)3Cl12·24H2O]	NaCl100%	140/89	4	m/f	BP and heart rate were measured using an automatic sphygmomanometer (Omron M4-I, fully automatic BP monitor, Omron Matsusaka Co, Ltd., Japan) following 10 min rest in a sitting position. BP was measured three times with intervals of at least two minutes, between the hours of 7:00 am and 12:00 noon. BP was measured using the non-dominant arm with the exception of the first study visit during which BP was measured using both arms. If the BP in the two arms differed in SBP or DBP by more than 10 mmHg, the arm with the higher reading was used for all subsequent measurements. Volunteers were not told the results of their BP measurements during the study and the study nurse was unaware of the treatment allocations
Yang (a)	2018	Single blind, randomized, controlled trial	China	24	27	67.8	6 months	Stage 2 of hypertension	Low sodium salt [Nacl65%, Kcl30%, Ca salts5% and 12 mg/kg folic acid]	NaCl100%	161/80.6		m/f	SBP and DBP were defined by Korotkoff sound phase 1 and phase 5, respectively.
Yang (b)	2018	Single blind, randomized, controlled trial	China	38	37	67.3	6 months	Stage 2 of hypertension	Low sodium salt [Nacl65%, Kcl30%, Ca salts5% and 12 mg/kg folic acid]	NaCl100%	159/85		m/f	SBP and DBP were defined by Korotkoff sound phase 1 and phase 5, respectively.
Zhao	2014	Patient-Blinded Randomized Controlled Trial	China	99	114	62.8	3 months	Stage 2 of hypertension	65% sodium chloride, 25%potassium chloride and 10% magnesium sulfate	NaCl100%	174.1/102.2	5	m/f	Blood pressure was measured with three consecutive blood pressure measurements (with at least one minute’s rest between each measurement) from a seated subjects’ right arm in a quiet room. A previously validated electronic sphygmomanometer (OMRON HEM-759P, Dalian China) was used.
Zhou	2009	Single-blind, randomized, controlled trial	China	62	64	67.5	6 months	Stage 2 of hypertension	NaCl, 65%; KCl, 30%; Calcium saits 5%; 12 mg/kg folic acid	NaCl100%	159.7/83.3	4	m/f	the measurement were performed by two experienced physicians
Zhou	2013	Double-blind, randomized controlled trial	China	224	238	46(Total)	24 months	Stage 2 of hypertension	NaCl, 65%; KCl, 25%; MgSO4, 10%	NaCl100%	154/91	5	m/f	Blood pressure was measured at each visit by trained doctors using an Omron HEM-770A automatic sphygmomanometer (OMRON (China) Co., Ltd. (OCE/OCE-HCB/OCE-SH), Shanghai, China). Blood pressure was measured in the right arm at the heart level in the seated position.

### Study quality

**Fig. 2:**
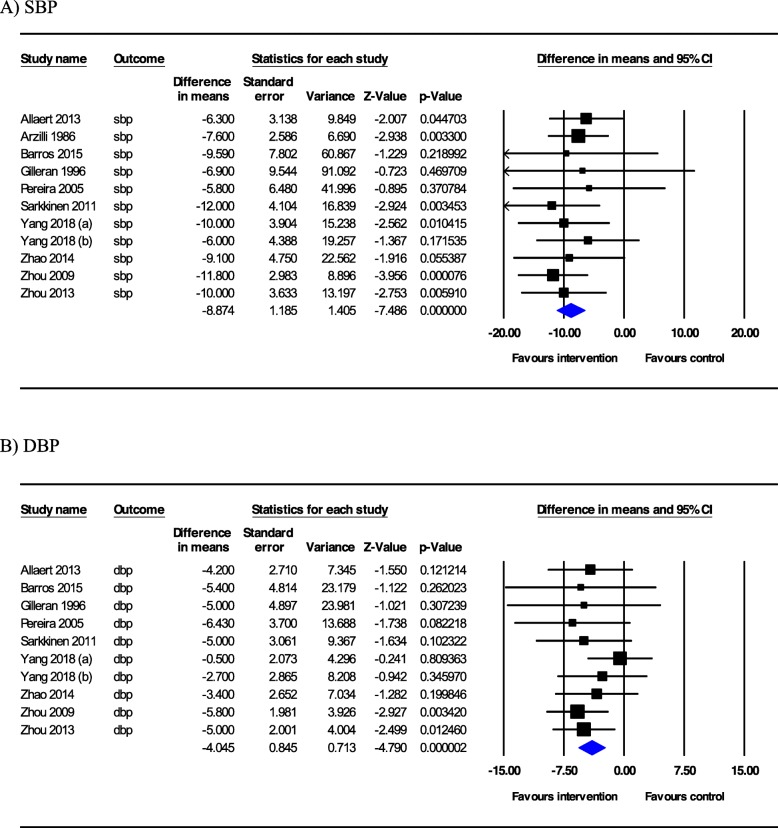
Forest plot of comparison of blood pressure between salt substitutive and control groups. **a** systolic blood pressure and (**b**) diastolic blood pressure. Random effects model was used to estimate the weighted mean differences of indicators. CI, confidence interval; I-squared inconsistency

Risk of bias assessments were performed for all 11 trials included in the meta-analysis and are summarized in Fig. 2. The Jadad score of these trials was relatively high, and most included trials had a score of more than 3. As it has been shown in previous studies, in which the scores of more than three are considered as high quality, six trials are categorized as high-quality studies [17, 21, 22, 24–26] and the remaining trials were divided into low quality studies [14, 20] and unclear quality studies [27, 28] (Table 2).

**Table 2:** Quality of the nine included studies by Jadad scale

Study; Year	Blinding	Randomization	Withdrawals and dropouts descriptions	Total Score
Allaert, 2013	1	1	1	3
Arzilli, 1986	Not clear	Not clear	Not clear	Not clear
Barros, 2015	2	2	0	4
Gilleran, 1996	Not clear	Not clear	Not clear	Not clear
Pereira, 2005	2	1	1	4
Sarkkinen, 2011	1	1	0	2
Yang(a), 2018	1	1	0	2
Yang(b), 2018	1	1	0	2
Zhao, 2014	2	2	1	5
Zhou, 2009	2	2	1	5
Zhou, 2013	2	2	1	5

### Effect of salt substitutes on blood pressure

The results of the meta-analysis, including the 11 trials, are presented in Fig. 2. Pooled weighted mean differences showed the significant reductions of SBP (WMD − 8.87 mmHg; 95% CI − 11.19, − 6.55, *p* < 0.001) and DBP (WMD − 4.04 mmHg; 95% CI − 5.70, − 2.39) values. No statistically significant heterogeneity was observed between the 11 included comparisons of SBPs and DBPs (I^2^ = 0%). However, we performed stratified analysis to further explore any potential effect of modulators on overall results.

### Stratified analysis

Subgroup analyses were carried out according to different modulators, including duration of intervention, mean age of participants in intervention groups, and quality of studies (Table 3). The duration of intervention was divided into < 3 months (shorter-term) and ≥ 3 months (longer-term). Another subgroup was based on the mean age of participants in intervention groups, in which trials were separated as adult (< 65 years old) and elderly (≥65 years old). Final subgroup analysis related to the quality of studies, in which two distinct categories were presented, as high-quality studies and low-quality studies. Significant reductions in SBP (7.93 mmHg), and DBP (5.02 mmHg) in shorter-term, and SBP (9.78 mmHg) and DBP (3.71 mmHg) in longer-term subgroups were observed after salt substitute intervention. The stratified analysis of trials based on the mean age of participants showed a significant reduction in the mean difference of SBP in both adult (< 65 years old) (WMD − 10.38 mmHg; 95% CI − 16.16, − 4.60) and elderly participants (≥65 years old) (WMD − 9.98 mmHg; (95% CI − 12.33, − 5.95)). However, the DBP-lowering effect of salt substitutes was only observed in adult (< 65 years old) patients (WMD − 4.22 mmHg; 95% CI − 7.85, − 0.58), but not in the elderly (≥65 years old) (WMD − 3.09 mmHg; 95% CI − 6.45, 0.27). After stratified analysis according to the study quality, both SBP and DBP were reduced by 9.14 mmHg (95% CI − 12.33, − 5.95) and 4.98 mmHg (95% CI − 7.05, − 2.91), respectively. However, the BP-lowering effect of salt substitute in low quality studies was only related to SBP (WMD: − 9.49 mmHg; (95% CI − 14.15, − 4.84)), but not DBP (WMD: − 2.13 mmHg; (95% CI − 5.02, 0.77)).

**Table 3:** Subgroup analysis

Subgroup*	WMD (95% CI)	Test for overall effect	Test for heterogeneity	I2(%)
Duration of study (weeks)				
< 3 months				
SBP	−7.93 [−11.23, −4.62]	*P* < 0.001	*P* = 0.84	0
DBP	−5.02 [−8.32, −1.72]	*P* = 0.003	*P* = 0.97	0
≥3 months				
SBP	−9.78 [−13.03, −6.53]	*P* < 0.001	*P* = 0.93	0
DBP	−3.71 [− 5.63, − 1.79]	*P* < 0.001	*P* = 0.53	0
Mean age of subjects				
< 65 years old				
*SBP*	−10.38 [− 16.16, −4.60]	*P* < 0.001	*P* = 0.83	0
*DBP*	−4.22 [−7.85, −0.58]	*P* = 0.02	*P* = 0.91	0
≥65 years old				
*SBP*	−9.98 [−14.06, −5.90]	*P* < 0.001	*P* = 0.55	0
*DBP*	−3.09 [−6.45, 0.27]	*P* = 0.07	*P* = 0.18	42
Quality of studies				
High quality				
*SBP*	−9.14 [−12.33, −5.95]	*P* < 0.001	*P* = 0.86	0
*DBP*	−4.98 [−7.05, −2.91]	*P* < 0.001	*P* = 0.98	0
Low quality				
*SBP*	−9.49 [−14.15, −4.84]	*P* < 0.001	*P* = 0.60	0
*DBP*	−2.13 [−5.02, 0.77]	*P* = 0.15	*P* = 0.47	0

*: Abbreviations: *SBP* Systolic blood pressure, *DBP* Diastolic blood pressure, *WMD* Weighted mean difference, *CI* Confidence interval, I2, percentage score for heterogeneity

### Sensitivity analysis

**Fig. 3:**
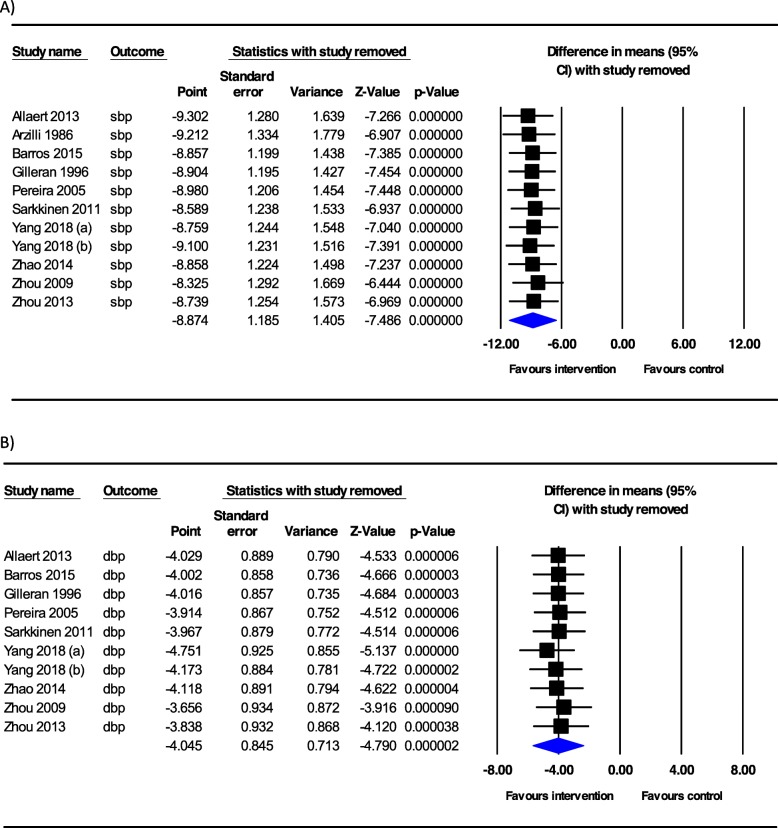
Sensitivity analysis for the effect of salt substitutes on (**a**) systolic blood pressure, **b** diastolic blood pressure

Sensitivity analysis was performed by removing each trial in turn and recalculating the pooled WMD. The systematic removal of each trial did not change the pooled effects of salt substitute on SBP and DBP values, which ranged from − 8.32 (95% CI = -10.85, − 5.79) to − 9.30 (95% CI = -11.81, − 6.79) in SBP, and − 3.65 (95% CI = -5.48, − 1.82) to − 4.75 (95% CI = -6.56, − 2.93) in DBP values (Fig. 3).

### Publication Bias

**Fig. 4:**
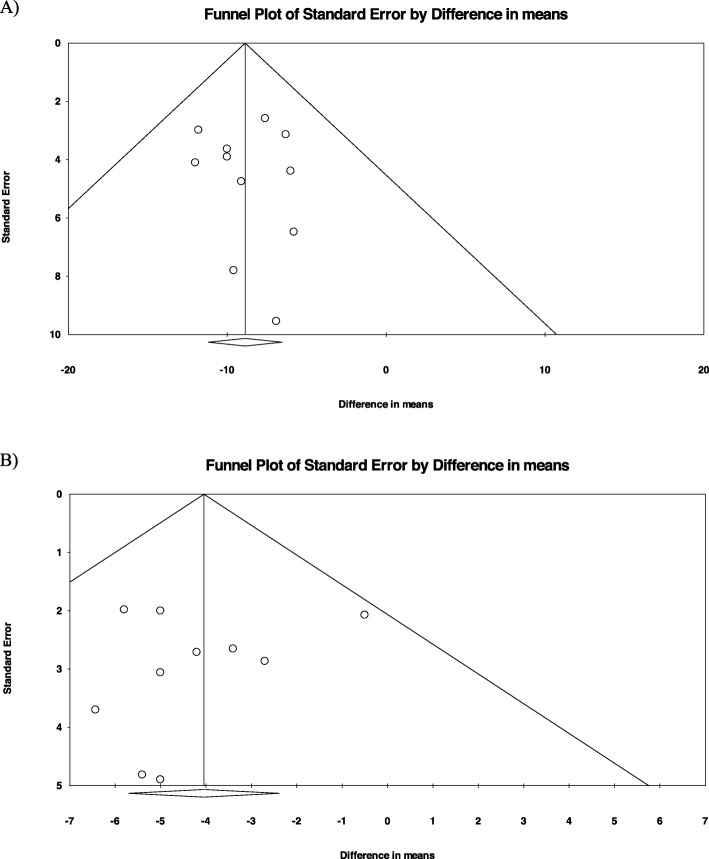
Funnel plot detailing publication bias of included studies according to (**a**) systolic blood pressure and (**b**) diastolic blood pressure. MD = Mean Difference, SE = standard error

Visual inspection of the funnel plots of standard error by effect size (WMD) of both SBP and DBP were symmetrical, indicating no potential publication biases in the present meta-analysis. Additionally, results from Egger’s linear regression [SBP (intercept: 0.13; standard error: 0.55; 95% CI: − 1.11, 1.39; t = 0.25, df = 9; two-tailed *p* = 0.80); DBP (intercept: − 0.53; standard error: 0.84; 95% CI: − 2.49, 1.42; t = 0.63, df = 8; two-tailed *p* = 0.54)] and Begg’s rank correlation test (SBP: Kendall’s Tau with continuity correction: 0.07; z = 0.31; two-tailed *p* = 0.75; DBP: Kendall’s Tau with continuity correction:0.00; z = 0.00; two-tailed *p* = 1.00) also suggested no evidence of potential publication bias **(**Fig. 4**)**.

## Discussion

In the present systematic review and meta-analysis, we found that supplementation with salt substitutes is associated with significant decreases in both SBP and DBP. Moreover, no significant heterogeneity was noted between the 11 included comparisons of SBP and DBP. Our results confirmed that both short and long-term use yielded significant decreases in SBP and DBP. In addition, stratified analysis showed a significant decrease in the mean difference of SBP in both adult (< 65 years old) and elderly (≥65 years old). However, the DBP-lowering impacts of salt substitutes was only found in adult patients, not in the elderly subjects.

HTN is an important risk factor for a wide range of diseases, including cardiovascular diseases, stroke, and various types of cancer. Despite concerted efforts, global access to effective pharmacological treatments for HTN remains challenging [29]. Salt substitutes have emerged as an adjunct or mono-therapy in the treatment of HTN and related diseases. Empirical evidence suggests that salt substitutes exert their pharmacological effects via targeting and affecting sequences of cellular and molecular processes. Results from empirical investigations suggest that salt intake plays a role in the development of HTN [30, 31]; whilst various RCTs have affirmed that simply decreasing salt intake could elicit hypotensive effects in both hypertensive and normotensive subjects [32, 33]. There are multiple mechanisms implicating the potential benefits of salt restriction in the diet; high salt consumption results in an increase in blood volume and peripheral vascular resistance, thus, limiting salt intake could facilitate a reduction in BP [34]. Additionally, salt restriction could lead to decreased production and infiltration of inflammatory markers, consequently lowering blood pressure [35]. Moreover, salt restriction has been suggested as an inhibiting factor in the production of ROS (reactive oxygen species), thus improving peripheral vascular resistance and subsequently reducing BP [36]. International guidelines now suggest a population-based salt restriction for the treatment and prevention of HTN [37, 38], whilst at the seminal United Nations Non-Communicable Disease Summit in 2011, the World Health Organization supported a reduction in dietary salt intake [39, 40].

The UK salt-reduction program is one of most influential programs worldwide, and has yielded significant reductions in the salt content of several processed foods through a food industry–level intervention [41]. Contrastingly, in developing countries, the main source of dietary sodium is from home cooking [42]. Hence, it is conceivable that salt-reduction approaches typically used in developed countries could be ineffective and inappropriate in the developing world, and other approaches must be assessed [41]. In the early 1990’s, a potassium- and magnesium-enriched salt alternative was introduced that effectively led to the reduced presence of sodium in foods [43]. Subsequently, several epidemiologic reports and clinical trials have investigated the hypotensive effects of salt substitutes [20, 22, 44].

Zhou et al., investigated the long-term impact of salt substitution (comprised of 65% sodium chloride, 10% magnesium sulfate, 25% potassium chloride) vs. normal salt (100% sodium chloride) on BP among 200 families in rural China [22]. Zhou et al., reported that SBP and DBP were significantly reduced in normotensive subjects, and SBP was reduced in hypertensive subjects. These findings suggest that salt substitution may be introduced as an efficacious adjuvant therapy for subjects with HTN, and may be used in the prevention of HTN in normotensive individuals [22]. In another report, Zhou and colleagues assessed the therapeutic effects of salt substitutes on subjects with HTN in a rural population of North China [23]. Participants were dichotomized into either a normal salt (100% sodium chloride) or low salt substitute (65% NaCl, 10% MgSO4, and 25% KCl), and the authors reported that the low sodium substitute led to a significant decrease in SBP and DBP, in comparison to regular salt. Moreover, participants aged 40–70 years had a greater response to the salt substitute than those aged < 40 or > 70 years, respectively; whilst the low salt substitute was associated with heterogenous benefits in both females and males.

Previous meta-analyses have investigated the effects of salt substitutes on blood pressure [10, 45]. Indeed, Peng et al., [10] documented that salt substitutes can elicit significant reductions in SBP (− 4.9 mmHg) and DBP (− 1.5 mmHg). In the present systematic review and meta-analysis, we provide greater clarity into the efficaciousness of salt substitutes for reducing blood pressure, where we were able to include a greater number of relevant studies (11 trials vs 6 trials), incorporated a larger sample size, had low heterogeneity, and we also used pre-specified subgroups to assess the impact of moderators. The overarching result remains the same as previous work, however, we reported greater reductions in both SBP (WMD − 8.87 mmHg; 95% CI − 11.19, − 6.55, *p* < 0.001) and DBP (WMD − 4.04 mmHg; 95% CI − 5.70, − 2.39), which is likely attributable to the increased number of studies included for meta-analysis.

Interestingly, a significant reduction in the mean difference of SBP in both adult (< 65 years old) and elderly populations (≥65 years old) was detected, whereas the DBP-lowering effect of salt substitutes was observed in adult (< 65 years old) patients, but not in the elderly (≥65 years old). Whilst both SBP and DBP are considered independent predictors of CVD in younger people, SBP could be considered as a suitable predictor for elderly subjects [46, 47]. Moreover, it is suggested that anti-hypertensive agents be more acutely considered in elderly persons, as although DBP was reduced to < 90 mmHg in 90% of subjects, SBP was lowered to < 140 mmHg in just 60% of elderly subjects [46, 47].

Salt substitutes have been principally shown to reduce urinary sodium excretion and increase potassium excretion in varying populations [24, 28]; we can therefore assert that the reduction in BP that we observed in this meta-analysis is likely attributable to the salt substitutes. Of note, whilst perception or palatability of saltiness was not investigated in this meta-analysis, it has been demonstrated in previous work [16] that supplementation with a salt-substitute does not alter, nor impact, the perception of saltiness, enjoyability, or overall acceptability of food. It has also been reported that salt sensitivity is more common among older individuals and significantly increases with age [48], which could be considered as a reason for between-studies differences. Salt sensitivity is a phenomenon which emphasizes the variation of BP among individuals in response to dietary salt intake [49], though the underlying mechanisms of the phenomenon remain unclear. However, there are some proposed mechanisms regarding the pathogenesis of salt sensitive HTN, including impairment in the renin angiotensin aldosterone system [50], renal trans-membrane sodium transport [50], the nitric oxide (NO) system, and the vascular endothelium [51]. Notwithstanding, recent studies indicate that modification of endothelial cell function and non-osmotic storage of salt could be the result of changes in endothelial surface layer characteristics. Therefore, it seems that, in addition to kidney malfunction, endothelial dysfunction could be considered as an effective factor related to salt sensitivity and sodium homeostasis [52].

There is some evidence demonstrating stronger BP reducing effects among elderly people vs young people [53], and women vs men [54], following a reduction in sodium intake. However, such discrepancies may be attributed, or indeed, counter-acted when accounting for baseline BP values and health status. Of note, some physiological studies have suggested that female hormones (estrogen and progesterone) might be associated with increased renal sodium reabsorption and water retention [55], and sex hormone genes variants have been strongly correlated with BP response to a dietary-sodium intervention [56]. Although there are differences regarding the responses to salt intake with regards to sex, we could not dichotomize subjects by sex due to insufficient reporting of BP and sex characteristics. Notwithstanding, such factors need more detailed investigation.

The current meta-analysis has various limitations, including the lack of full-text access to some articles, low sample size, and that the clinical symptoms of the cases were varied. Further consideration must be taken should clinicians wish to advocate salt substitutes to patients with multiple co-morbidities; where adverse effects of salt substitutes in patients with severe renal impairment have been reported [57, 58] and should, therefore, be considered when applying salt-substitution strategies in large and diverse populations. Moreover, five trials (of 11 included trials) were conducted in China, which limits the generalizability of the results. Although there were no specific heterogeneities between studies, variation in study duration, which ranged from 8 days to 24 months, age of subjects, ranging from 39.5 and 67.8 years old, and varying patterns of drugs administration could influence the overall results and should be considered as another limitation of the present study. The inability to conduct subgroup analysis based on sex, due to insufficiency in the available data, could be considered as another limitation of the present study, but was out of the operational control of the study.

Notwithstanding the limitations, the present study has numerous strengths; we have analyzed a reasonable number of studies (11 trials), included a considerable sample size, reported low heterogeneity, and we also used pre-specified subgroups to evaluate the effects of modulators.

## Conclusions

In conclusion, the results of our meta-analysis highlight that salt substitutes are an efficacious supplement for the lowering of SBP and DBP in patients with stage 2 HTN. Thus, it is conceivable that salt substitution may be a feasible dietary approach for population-level control of HTN. Moreover, given that HTN has been ranked the number one risk factor, globally, associated with the burden of disease [59], identifying appropriate and sustainable, cultural and economic interventions with long-term effectiveness is vitally important. Whilst salt substitution shows promise, a greater number of studies conducted using large and diverse samples, are required to affirm their efficacy at the population level.

## Data Availability

We incorporated only peer-reviewed, published articles. The datasets (as derived from the published papers) used and analyzed during the current study are available on reasonable request from the corresponding author.

## Abbreviations

BP: Blood pressure
CI: Confidence interval
CTS: Cardiotonic steroids
DBP: Diastolic blood pressure
HTN: Hypertension
PRISMA: Preferred reporting items for systematic reviews and meta-analyses
RCT: Randomized controlled trials
SBP: Systolic blood pressure
WMD: Weighted mean difference

## References

[1] Mancia G, De Backer G, Dominiczak A, Cifkova R, Fagard R, Germano G, Grassi G, Heagerty AM, Kjeldsen SE, Laurent S (2007). 2007 guidelines for the Management of Arterial Hypertension: the task force for the Management of Arterial Hypertension of the European Society of Hypertension (ESH) and of the European Society of Cardiology (ESC). J Hypertens.

[2] Murray CJ, Lopez AD (1997). Global mortality, disability, and the contribution of risk factors: global burden of disease study. Lancet.

[3] Kearney PM, Whelton M, Reynolds K, Muntner P, Whelton PK, He J (2005). Global burden of hypertension: analysis of worldwide data. Lancet.

[4] Cicila GT, Rapp JP, Wang JM, St Lezin E, Ng SC, Kurtz TW (1993). Linkage of 11 beta-hydroxylase mutations with altered steroid biosynthesis and blood pressure in the Dahl rat. Nat Genet.

[5] Joe B, Shapiro JI (2012). Molecular mechanisms of experimental salt-sensitive hypertension. J Am Heart Assoc.

[6] Fedorova OV, Shapiro JI, Bagrov AY (2010). Endogenous cardiotonic steroids and salt-sensitive hypertension. Biochim Biophys Acta.

[7] Ong KL, Tso AW, Lam KS, Cheung BM (2008). Gender difference in blood pressure control and cardiovascular risk factors in Americans with diagnosed hypertension. Hypertension.

[8] Lionakis N, Mendrinos D, Sanidas E, Favatas G, Georgopoulou M (2012). Hypertension in the elderly. World J Cardiol.

[9] Havas S, Dickinson BD, Wilson M (2007). The urgent need to reduce sodium consumption. Jama.

[10] Peng YG, Li W, Wen XX, Li Y, Hu JH, Zhao LC (2014). Effects of salt substitutes on blood pressure: a meta-analysis of randomized controlled trials. Am J Clin Nutr.

[11] Moher D, Liberati A, Tetzlaff J, Altman DG (2009). Preferred reporting items for systematic reviews and meta-analyses: the PRISMA statement. Ann Intern Med.

[12] Whelton PK, Carey RM, Aronow WS, Casey DE, Collins KJ, Himmelfarb CD, DePalma SM, Gidding S, Jamerson KA, Jones DW (2018). 2017 ACC/AHA/AAPA/ABC/ACPM/AGS/APhA/ASH/ASPC/NMA/PCNA guideline for the prevention, detection, evaluation, and management of high blood pressure in adults: a report of the American College of Cardiology/American Heart Association task force on clinical practice guidelines. J Am Coll Cardiol.

[13] Follmann D, Elliott P, Suh I, Cutler J (1992). Variance imputation for overviews of clinical trials with continuous response. J Clin Epidemiol.

[14] Yang G-H, Zhou X, Ji W-J, Liu J-X, Sun J, Shi R, Jiang T-M, Li Y-M (2018). Effects of a low salt diet on isolated systolic hypertension: A community-based population study. Medicine.

[15] Pt Higgins J, Green S (2009). Cochrane Handbook for systematic reviews of interventions, vol. 5.

[16] China Salt Substitute Study Collaborative Group (2007). Salt substitution: a low-cost strategy for blood pressure control among rural Chinese. A randomized, controlled trial. J Hypertens.

[17] Barros CL, Sousa AL, Chinem BM, Rodrigues RB, Jardim TS, Carneiro SB, Souza WK, Jardim PC (2015). Impact of light salt substitution for regular salt on blood pressure of hypertensive patients. Arq Bras Cardiol.

[18] Geleijnse JM, Witteman JC, Bak AA, den Breeijen JH, Grobbee DE (1994). Reduction in blood pressure with a low sodium, high potassium, high magnesium salt in older subjects with mild to moderate hypertension. Bmj.

[19] Hu J, Zhao L, Thompson B, Zhang Y, Wu Y (2018). Effects of salt substitute on home blood pressure differs according to age and degree of blood pressure in hypertensive patients and their families. Clin Exp Hypertens.

[20] Sarkkinen ES, Kastarinen MJ, Niskanen TH, Karjalainen PH, Venalainen TM, Udani JK, Niskanen LK (2011). Feasibility and antihypertensive effect of replacing regular salt with mineral salt -rich in magnesium and potassium- in subjects with mildly elevated blood pressure. Nutr J.

[21] Zhao X, Yin X, Li X, Yan LL, Lam CT, Li S, He F, Xie W, Sang B, Luobu G (2014). Using a low-sodium, high-potassium salt substitute to reduce blood pressure among Tibetans with high blood pressure: a patient-blinded randomized controlled trial. PLoS One.

[22] Zhou B, Wang HL, Wang WL, Wu XM, Fu LY, Shi JP (2013). Long-term effects of salt substitution on blood pressure in a rural north Chinese population. J Hum Hypertens.

[23] Zhou B, Webster J, Fu LY, Wang HL, Wu XM, Wang WL, Shi JP (2016). Intake of low sodium salt substitute for 3years attenuates the increase in blood pressure in a rural population of North China - a randomized controlled trial. Int J Cardiol.

[24] Zhou X, Liu JX, Shi R, Yang N, Song DL, Pang W, Li YM (2009). Compound ion salt, a novel low-sodium salt substitute: from animal study to community-based population trial. Am J Hypertens.

[25] Pereira MAG, Galvão R, Zanella MT (2005). Effects of potassium supplementation by salt on arterial blood pressure and insulin resistance in hypertensive obese patients on diuretic therapy. Rev Nutr.

[26] Allaert F (2013). Double-blind, randomized, crossover, controlled clinical trial of NaCl+ chitosan 3% versus NaCl on mild or moderate high blood pressure during the diet and lifestyle improvement period before possible prescription of an antihypertensive treatment. Int Angiol.

[27] Arzilli F, Taddei S, Graziadei L, Bichisao E, Giovannetti R, Salvetti A (1986). Potassium-rich and sodium-poor salt reduces blood pressure in hospitalized patients. J Hypertension Suppl.

[28] Gilleran G, O'leary M, Bartlett W, Vinall H, Jones A, Dodson P (1996). Effects of dietary sodium substitution with potassium and magnesium in hypertensive type II diabetics: a randomised blind controlled parallel study. J Hum Hypertens.

[29] Palafox B, McKee M, Balabanova D, AlHabib KF, Bahonar A, Ismail N, Chifamba J, Chow CK, Corsi DJ, Dagenais GR (2016). Wealth and cardiovascular health: a cross-sectional study of wealth-related inequalities in the awareness, treatment and control of hypertension in high-, middle-and low-income countries. Int J Equity Health.

[30] Law MR, Frost CD, Wald NJ (1991). By how much does dietary salt reduction lower blood pressure? III--analysis of data from trials of salt reduction. Bmj.

[31] Elliott P (1991). Observational studies of salt and blood pressure. Hypertension.

[32] Strazzullo P, D'Elia L, Kandala NB, Cappuccio FP (2009). Salt intake, stroke, and cardiovascular disease: meta-analysis of prospective studies. Bmj.

[33] He FJ, Li J, Macgregor GA (2013). Effect of longer term modest salt reduction on blood pressure: Cochrane systematic review and meta-analysis of randomised trials. Bmj.

[34] Machnik A, Neuhofer W, Jantsch J, Dahlmann A, Tammela T, Machura K, Park J-K, Beck F-X, Müller DN, Derer W (2009). Macrophages regulate salt-dependent volume and blood pressure by a vascular endothelial growth factor-C–dependent buffering mechanism. Nat Med.

[35] Chan CT, Moore JP, Budzyn K, Guida E, Diep H, Vinh A, Jones ES, Widdop RE, Armitage JA, Sakkal S (2012). Reversal of vascular macrophage accumulation and hypertension by a CCR2 antagonist in deoxycorticosterone/salt-treated mice. Hypertension.

[36] Boegehold MA (2013). The effect of high salt intake on endothelial function: reduced vascular nitric oxide in the absence of hypertension. J Vasc Res.

[37] Muntner P, Krousel-Wood M, Hyre AD, Stanley E, Cushman WC, Cutler JA, Piller LB, Goforth GA, Whelton PK (2009). Antihypertensive prescriptions for newly treated patients before and after the main antihypertensive and lipid-lowering treatment to prevent heart attack trial results and seventh report of the joint national committee on prevention, detection, evaluation, and treatment of high blood pressure guidelines. Hypertension.

[38] 2018 Practice Guidelines for the management of arterial hypertension of the European Society of Hypertension and the European Society of Cardiology: ESH/ESC Task Force for the Management of Arterial Hypertension: Erratum. J Hypertens. 2019;37(2):456. 10.1097/HJH.0000000000002026 30640882

[39] Organization WH (2011). Strategies to monitor and evaluate population sodium consumption and sources of sodium in the diet: report of a joint technical meeting convened by WHO and the Government of Canada.

[40] Organization WH: United Nations high-level meeting on noncommunicable disease prevention and control. http. In. 2015.

[41] He FJ, Pombo-Rodrigues S, Macgregor GA (2014). Salt reduction in England from 2003 to 2011: its relationship to blood pressure, stroke and ischaemic heart disease mortality. BMJ Open.

[42] Anderson CA, Appel LJ, Okuda N, Brown IJ, Chan Q, Zhao L, Ueshima H, Kesteloot H, Miura K, Curb JD (2010). Dietary sources of sodium in China, Japan, the United Kingdom, and the United States, women and men aged 40 to 59 years: the INTERMAP study. J Am Diet Assoc.

[43] Mervaala EM, Himberg JJ, Laakso J, Tuomainen P, Karppanen H (1992). Beneficial effects of a potassium- and magnesium-enriched salt alternative. Hypertension.

[44] Jeffery RW, Pirie PL, Elmer PJ, Bjornson-Benson WM, Mullenbach VA, Kurth CL, Johnson SL (1984). Low-sodium, high-potassium diet: feasibility and acceptability in a normotensive population. Am J Public Health.

[45] Hernandez AV, Emonds EE, Chen BA, Zavala-Loayza AJ, Thota P, Pasupuleti V, Roman YM, Bernabe-Ortiz A, Miranda JJ (2019). Effect of low-sodium salt substitutes on blood pressure, detected hypertension, stroke and mortality. Heart.

[46] Einhorn PT, Davis BR, Massie BM, Cushman WC, Piller LB, Simpson LM, Levy D, Nwachuku CE, Black HR, Group ACR (2007). The antihypertensive and lipid lowering treatment to prevent heart attack trial (ALLHAT) heart failure validation study: diagnosis and prognosis. Am Heart J.

[47] Black HR, Elliott WJ, Grandits G, Grambsch P, Lucente T, White WB, Neaton JD, Grimm RH, Hansson L, Lacourcière Y (2003). Principal results of the controlled onset verapamil investigation of cardiovascular end points (CONVINCE) trial. Jama.

[48] Weinberger MH, Fineberg NS (1991). Sodium and volume sensitivity of blood pressure. Age and pressure change over time. Hypertension.

[49] Kawasaki T, Delea CS, Bartter FC, Smith H (1978). The effect of high-sodium and low-sodium intakes on blood pressure and other related variables in human subjects with idiopathic hypertension. Am J Med.

[50] Giner V, Poch E, Bragulat E, Oriola J, González D, Coca A, de la Sierra A (2000). Renin-angiotensin system genetic polymorphisms and salt sensitivity in essential hypertension. Hypertension.

[51] Bragulat E, de la Sierra A, Antonio MT, Coca A (2001). Endothelial dysfunction in salt-sensitive essential hypertension. Hypertension.

[52] Choi HY, Park HC, Ha SK (2015). Salt sensitivity and hypertension: a paradigm shift from kidney malfunction to vascular endothelial dysfunction. Electrolytes Blood Pressure.

[53] Vollmer WM, Sacks FM, Ard J, Appel LJ, Bray GA, Simons-Morton DG, Conlin PR, Svetkey LP, Erlinger TP, Moore TJ (2001). Effects of diet and sodium intake on blood pressure: subgroup analysis of the DASH-sodium trial. Ann Intern Med.

[54] He J, Gu D, Chen J, Jaquish CE, Rao DC, Hixson JE, Chen J-c, Duan X, Huang J-f, Chen C-S (2009). Gender difference in blood pressure responses to dietary sodium intervention in the GenSalt study. J Hypertens.

[55] Stachenfeld NS, Taylor HS (2004). Effects of estrogen and progesterone administration on extracellular fluid. J Appl Physiol.

[56] Kelly TN, Rebholz CM, Gu D, Hixson JE, Rice TK, Cao J, Chen J, Li J, Lu F, Ma J (2013). Analysis of sex hormone genes reveals gender differences in the genetic etiology of blood pressure salt sensitivity: the GenSalt study. Am J Hypertens.

[57] Ray K, Dorman S, Watson R (1999). Severe hyperkalaemia due to the concomitant use of salt substitutes and ACE inhibitors in hypertension: a potentially life threatening interaction. J Hum Hypertens.

[58] van der Loeff HS, van Schijndel RS, Thijs L (1988). Cardiac arrest due to oral potassium intake. Intensive Care Med.

[59] Lim SS, Vos T, Flaxman AD, Danaei G, Shibuya K, Adair-Rohani H, AlMazroa MA, Amann M, Anderson HR, Andrews KG (2012). A comparative risk assessment of burden of disease and injury attributable to 67 risk factors and risk factor clusters in 21 regions, 1990–2010: a systematic analysis for the global burden of disease study 2010. Lancet.

